# Case of Pretended Mayhem, Which Was Tried before the Mayor’s Court in Cincinnati, March, 1840

**Published:** 1841-05

**Authors:** S. D. Gross


Art. V—*Case of Pretended Mayhem, which was tried before the Mayor's Court in Cincinnati, March,* 1840. By S. D, Gross, M. D.

The following case is interesting not only in a physiological point of view, but also as a "little piece" of medical jurisprudence, and deserves to be recorded as a warning to those who, under an idea that they are infallible, never make any but the most hasty and imperfect examinations, no matter whether they involve life, property, or reputation.

Two lads, Hiram and Jackson Black, the one fourteen, the other eleven years of age, after having resided about two years at the Shaker village, in Crosby Township, within twenty-two miles of Cincinnati, returned to their home in Bracken county, Kentucky, where a report was soon after circulated that they had been emasculated by some of their late brethren. Upon inquiry, the mother and uncle of the boys confirmed the story, alleging that both were without testes, in consequence of an operation that had been performed on them the preceding summer. An outrage such as this was well calculated to inflame the public mind, and accordingly several of the most intelligent and influential citizens of the neighborhood, determined to sift the matter to the bottom, addressed a letter to Bellamy Storer, Esq., requesting him forthwith to institute suit against the supposed offenders. The boys, accompanied by their uncle, soon after appeared in Cincinnati, making affidavit to the circumstance above mentioned, and undergoing an examination by an old medical practitioner, who, in addition to the deficiencies already alluded to, testified as to the presence of distinct and well-marked cicatrices on the scrotum of each of them.

In consequence of the evidence of this gentleman, legal proceedings were immediately instituted against the Shakers, four of whom were apprehended, and lodged in the county jail. In the meanwhile, at the request of the counsel for the defendants, Messrs. Riddle & Roll, I visited the village to examine into the condition of the other male children at the establishment, with a view of ascertaining whether they also had been subjected to that kind of treatment. It was soon apparent, however, that no such operation had been performed, as all the boys, twelve in number, from the age of two years up to that of eighteen, had no cause of complaint in that particular.

The next day, after reporting the result of my observations to the court, I was requested also to examine the complainants, Hiram and Jackson Black, in conjunction with the gentleman who had previously investigated their cases, and testified as to the absence of their testes. The scrotum of each was, indeed, empty, but as to scars or cicatrices, none could be seen, though several folds of the skin, or rather the smooth, glossy intervals between them, were pointed out, with no little self-complacency, as evidences of their existence. On a little further search the *lost* testicles were found to be situated in the groin, a little below the external ring, whence with a little traction they could be easily forced down into the scrotum. This announcement of course put at once an end to the trial, to the great delight of the poor Shakers, the dismay of the *medical jurist*, and the great merriment of the by-standers.

It is not a little singular that, in both these cases, the testes should have been so late in descending into the scrotum. The lads were of rather short stature for their age, but in other respects perfectly well-formed. The testes were about the ordinary size and consistence; and although they could be easily pressed, as already stated, into the scrotum, yet they immediately re-ascended to their former position, as soon as the fingers were removed.

